# Aflatoxins and *A. flavus* Reduction in Loaf Bread through the Use of Natural Ingredients

**DOI:** 10.3390/molecules23071638

**Published:** 2018-07-04

**Authors:** Juan M. Quiles, Raquel Torrijos, Fernando B. Luciano, Jordi Mañes, Giuseppe Meca

**Affiliations:** 1 Laboratory of Food Chemistry and Toxicology, Faculty of Pharmacy, University of Valencia, Av. Vicent Andrés Estellés s/n, 46100 Burjassot, Spain; juan.quiles@uv.es (J.M.Q.); tocara@alumni.uv.es (R.T.); jorge.manes@uv.es (J.M.); 2 School of AgriculturalSciences and Veterinary Medicine, PontifíciaUniversidade Católica do Paraná, BR 376 Km 14, 83010-500 São José dos Pinhais, Brazil; fernando.luciano@pucpr.br

**Keywords:** aflatoxins, shelf life, mustard flour, mycotoxin reduction, LC-MS/MS

## Abstract

In this study, the antifungal activity of yellow mustard (YMF) and oriental mustard (OMF) meal extracts against 14 strains of fungi was tested on a solid medium. The results obtained with the YMF were next confirmed in liquid medium determining the minimum inhibitory concentration (MIC) and the minimum fungicide concentration (MFC). Finally, the use of YMF as a natural preservative to extend the useful life of bread was evaluated. Breads with different concentrations of YMF (2, 4, 6 and 8 g/kg) were prepared and contaminated with *Aspergillus flavus* ISPA 8111 and *Penicillium nordicum* CECT 2320. For 10 days the formation of mycelium was observed, and after that the fungal growth and the mycotoxins production was determined. The results obtained with the YMF were compared with breads treated with the commercial additive sodium propionate (E-281). The results showed a significant reduction of the fungal population using 6 g/kg and 8 g/kg of YMF in bread contaminated with *A. flavus* and with *P. nordicum* and an extensions of the breads shelf life of 7 and 5 days, respectively, in comparison with the control experiment. A reduction of 78% of AFB_1_ was observed using 6 g/kg of YMF while no AFB_1_ production was detected employing 8 g/kg of YMF in bread preparation.

## 1. Introduction

**Figure 1:**
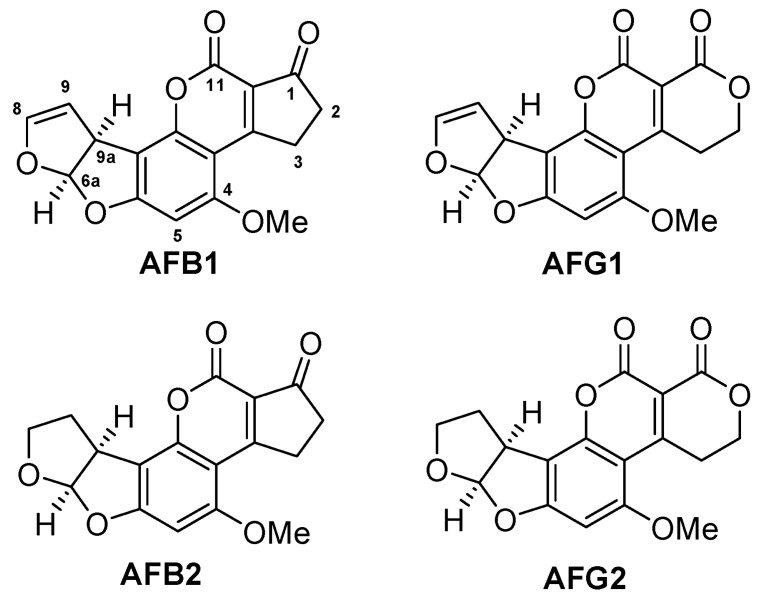
Chemical structures of the aflatoxins.

Aflatoxins (AFs) (Figure 1) are difuranocoumarin derivatives mainly produced through the polyketide pathway by two species of *Aspergillus* fungi which are especially found in areas with hot and humid climates. *Aspergillus flavus* is ubiquitous in Nature, preferring the colonization of the aerial parts of the plants (leaves, flowers) and usually producing group B AFs. *Aspergillus parasiticus* which produces both B and G AFs, is more adapted to soil environments and has more limited distribution [1]. *Aspergillus bombycis*, *Aspergillus ochraceoroseus*, *Aspergillus nomius*, and *Aspergillus pseudotamari* are also AF-producing species, but they are found less frequently [2].

The four main AFs are AFB_1_, AFB_2_, AFG_1_ and AFG_2_. They can directly contaminate agricultural products and other foodstuffs under pre-and post-harvest conditions. AFB_1_ is usually predominant in crops as well as in food products and it has been found to exhibit the greatest toxigenic potential [3]. The AFs can be classified from highest to lowest toxicity in the following order: AFB_1_, AFG_1_, AFB_2_ and AFG_2_, and this is probably explained by the presence of epoxidation at the 8,9 double bond, as well as by the greater power that accompanies the ring. AFB_1_ is one of the most potent toxic carcinogens, it is a teratogen and a mutagen and it is listed as a Group I carcinogen by the International Agency for Research on Cancer (IARC) because it is a cause of human primary hepatocellular carcinoma [4].

Plants belonging to the *Brassicaceae* family are known worldwide for their rich bioactive composition, highlighted by glucosinolates, which are cleaved by an enzymatic reaction to give isothiocyanates (ITCs). These ITCs possess many properties, among which their biocidal activity (fungicidal, bactericidal and on insects and small invertebrates), as well as herbicidal, antioxidant and anticancer effects may be highlighted [5]. Both *Sinapis alba* (yellow or white mustard) and *Brassica juncea* (brown and oriental mustard) contain high levels of glucosinolates, which are cleaved by myrosinase (EC 3.2.1.147) in the presence of moisture, forming ITCs plus thiocyanates, nitriles and some other minor compounds. In oriental mustard, myrosinase forms allyl isothiocyanate (AITC) from the main glucosinolate, sinigrin. Its effectiveness inhibiting the development of fungi [6,7], as well as the bacteria *Escherichia coli* O157: H7 [8], *Listeria monocytogenes* [9] or *Salmonella* sp. [10] has been proven.

In yellow mustard, the ITC that is formed is *p*-hydroxybenzylisothiocyanate (*p*-HBIT) from the main glucosinolate sinalbine. Ekanayake et al. [11] demonstrated that *p*-HBIT had significant antimicrobial activity against several foodborne pathogens, including *Escherichia coli*, *Staphylococcus aureus*, *Campylobacter jejuni*, *Pseudomonas aeruginosa*, *Salmonella enteritidis*, *Listeria monocytogenes*, *Shigella boydii* and *Clostridium* spp.

The aim of the study was to evaluate the use of oriental and yellow mustard flours to reduce contamination by toxigenic fungi and their mycotoxins. First, the antifungal activity of yellow mustard flour (YMF) and oriental mustard meal (OMF) was studied against fourteen different fungal species. Next, the reduction of fungal growth, the formation of mycotoxins and the YMF improvement of the useful life of moldy bread contaminated with *A. flavus* and *P. nordicum* were analyzed.

## 2. Results and Discussion

### 2.1. YMF and OMF Antifungal Activity

Table 1 shows the antifungal activity in solid medium evidenced by the eight different extracts tested (YMF and OMF, without and with heat treatment, directly and after concentration) on the 14 mycotoxigenic fungi used in this study.

**Figure 2:**
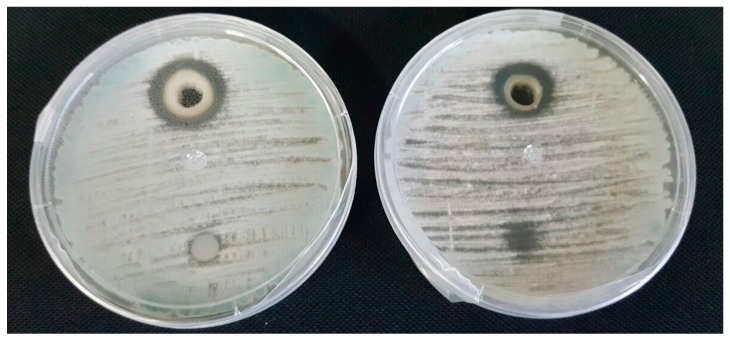
Antifungal activity of YMF lyophilized water extract against *P. nordicum* (CECT 2320) on PDA medium.

The non-autoclaved YMF was active only against *A. parasiticus*, whereas the concentrated extract of the non-autoclaved YMF showed the highest antifungal activity on the strains tested, and in particular on 13 of the 14 fungi studied (Figure 2). The autoclaved YMF extract was negative against all fungal strains tested, while the concentrated YMF extract was active against *P. camemberti* and *P. roqueforti*.

Considering the results of the antifungal activity of the OMF, only the concentrated extract of the non-autoclaved flour showed antifungal activity, against the strains of *P. roqueforti* and *A. carbonarius*. The other extracts of this bioactive ingredient tested did not shown any antifungal activity on the fungi tested at the incubation time employed. Considering the results of the antimicrobial activity on solid medium evidenced by the two matrices, the MIC and MFC of the concentrated extract of the no-autoclaved YMF was determined using the 96-well microplate assay.

As evidenced in Table 2, the MIC of the concentrated extract of YMF (non-autoclaved) ranged from 238.2 (*P. camemberti*) to 15,000 μg/mL (*A. flavus, A. parasiticus* and *A. carbonarius*). Important results of the antifungal activity of the YMF were also evidenced against *P. roqueforti* and *P. digitatum* with MIC data of 476.5 and 937.5 µg/mL respectively. Considering the MFC data, the YMF presented inhibition data that ranged from 1875 (*P. nordicum*, *P. commune* and *P. brevicompactum*) to 15,000 µg/mL (*A. flavus*, *A. parasiticus* and *A. carbonarius).* In general, the *Aspergillus* strains tested were more resistant to the YMF extract in comparison with the employed strains of *Penicillium* and *Fusarium*. Considering the *Penicillium* strains tested, the microorganisms that showed a very low resistance to the mustard extracts tested in this study were, *P. camemberti* and *P. roqueforti*, that showed the lower MIC values.

Considering the other *Penicillium* strains, the MIC values detected for the YMF were always above 500 µg/mL. The lyophilized extract of the YMF was also active against the strain of *F. verticillioides*, a fumonisins (FBs) producer, showing MIC and MFC values of 1875.5 and 3750.5 µg/mL, respectively.

Several authors have tested the application of the ITCs as antimicrobial substances, both directly and using mustard flours. In the first case, Mañes et al. [12] studied the antifungal activity of allyl isothiocyanate (AITC) against two mycotoxigenic strains of the genera *Aspergillus* and *Penicillium*. Tests of antifungal activity in a solid medium showed that 5 µL of AITC deposited inside a disc of sterile filter paper were sufficient to inhibit the growth of the *A. parasiticus* fungus seeded on the surface of a PDA medium plate. The amount of AITC needed to inhibit the growth of the fungus *P. expansum* was greater, specifically 25 μL/L. Azaiez et al. [13] evaluated the antifungal activity of AITC, phenyl (PITC) and benzyl isothiocyanates (BITC) toward *Fusarium* mycotoxigenic strains. The ITCs employed in the study inhibited the growth of three mycotoxigenic *Fusarium* (*Gibberella moniliformis*), reducing 2.1–89.7% of the mycelium size depending on the time and the dose used (from 10 to 50 µL). The activity of ITCs against non-fungal pathogenic microorganisms transmitted by food has also been studied. Ekanayake et al. [11] demonstrated that ρ-HBIT had significant bactericidal activity against *E. coli*, *S. aureus*, *C. jejuni*, *P. aeruginosa*, *S. enteritidis*, *L. monocytogenes*, *S. boydii* and *C. perfringens* at a dose of 0.35–2.13 mM.

Other studies have demonstrated the antimicrobial capacity of mustard flours directly instead of ITCs. Kanemaru and Miyamoto [14] compared the antimicrobial effects of mustard and purified AITC at equal concentrations of AITC. They found that mustard was more effective against *E. coli* than purified AITC. They found that 0.1% mustard with 9.4 µg/mL of AITC was able to inhibit the growth of *E. coli* in culture medium within 24 h but 12.3 µg/mL of purified AITC was required to achieve the same level of inhibition. Mayerhauser [15] found that retail style mustards eliminated 6 log10 of *E. coli* O157:H7 from trypticase soy broth within a few hours at refrigerator or room temperature. More recently, Rhee et al. [16] showed that mustard flour alone or with acetic acid reduced 6 log10 of *E. coli* O157:H7 to 0.3 log10 in 24 h at room temperature.

### 2.2. Inhibition of Fungal Growth

**Figure 3:**
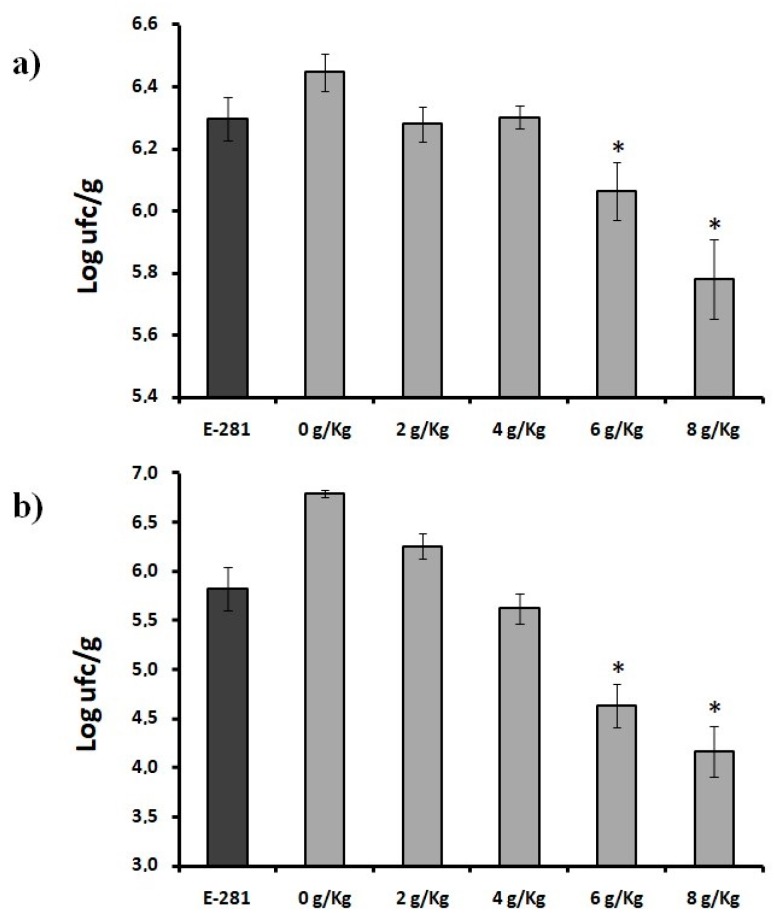
Population of (**a**) *A. flavus* and (**b**) *P. nordicum* in bread loaves treated with natural (YMF) and synthetic commercial (E-281) preservatives. Significantly different from the commercial control, *p* ≤ 0.05 (*), *p* ≤ 0.001 (**), *p* ≤ 0.0001 (***).

The growing demand for safe foods without synthetic chemical preservatives has prompted scientists to investigate the effects of natural compounds against the growth of several pathogenic microorganisms [8,17,18]. The evaluation of the use of YMF in breads inoculated with *A flavus* and *P. nordicum* compared with breads produced with sodium propionate, the classical commercial additive for bakery products, showed that none of the treatments used completely reduced the *A. flavus* and *P. nordicum* growth (Figure 3a,b). However, some preservative treatments applied in this study were able to reduce the fungal growth of the mycotoxigenic fungi tested in comparison to the control experiment.

As shown in Figure 3a, the bread contaminated with *A. flavus*, stored during 10 days and produced with sodium propionate presented a fungal contamination of 630 Log/UFC/g and no statistically differences in the microorganism growth were observed in the bread treated with 2 and 4 g/kg of YMF (628 and 630 Log/UFC/g respectively). In the bread treated with 6 g/kg (606 Log/UFC/g) and 8 g/kg (578 Log/UFC/g) a significant difference on the *A. flavus* was observed in comparison with the bread treated with the E-281, with a reduction of 0.24 and 0.52 Log/UFC/g respectively (47 and 67% UFC/g, respectively). 

Related to the results of the *P. nordicum* growth in the loaf bread produced with sodium propionate and with the YMF, the results of the fungal growth along the incubation period are shown in Figure 3b. In particular the bread loaves produced with sodium propionate (at 10 days storage) presented a fungal contamination of 582 Log UFC/g, whereas in the bread loaves treated with 2 and 4 YMF, the level of contamination was 626 and 562 Log UFC/g respectively. 

The bread treated with 6 and 8 g/kg of the YMF presented a statistically difference of *P. nordicum* growth of 463 and 416 Log UFC/g respectively in comparison with the bread produced with the E-281, with a reduction of the fungal growth of the 1.19 and 1.66 Log/UFC/g (94 and 97% UFC/g respectively). 

The use of ITCs generated from mustard flours has been studied by several authors. Quiles et al. [19] tested active packaging devices containing AITC or OMF + water to inhibit the growth of *A. parasiticus* in fresh pizza doughs after 30 days of inoculation. The antimicrobial activities were compared with a control group (non-antimicrobial treatment) and a group added with a commercial preservative (sodium propionate). The growth of *A. parasiticus* was inhibited after 30 days with AITC on filter paper at 5 µL/L and 10 µL/L, and on of OMF at 850 mg + 1 mL of water. The use of yellow mustard as an ingredient has been reported for acid food matrices against pathogenic microorganisms. Graumann and Holley [20] demonstrated that the *p*-HBITC generated in situ by including ground yellow mustard powder as an ingredient in dry-fermented sausages inhibited the growth of *E. coli* O157:H7.

### 2.3. Mycotoxin Reduction

Mean recovery of fortified bread loaves samples (n = 3) at three different levels of AFB_1_ and AFB_2_ (5, 10 and 15 μg/kg) was of 84.6 ± 3.6% and 88.2 ± 3.3%, respectively. The values obtained for recovery and relative standard deviations of the method used agree with the EU Commission Directive 2006/401/EC for methods to analyze bioactive compounds in foodstuffs [21]. Intra-day (n = 5) and inter-day (5 different days) variation values were 2.5 and 8.6%, respectively. These values are below 15%, which is the maximum variation for certification exercises of bioactive compounds. The detection limit (LOD) and the limit of quantification (LOQ) values were 0.05 and 0.15 μg/kg, respectively. Linearity, plotted as DAD response area against concentration estimated for the matrix matched standards, and matrix effects were studied using standard solutions and matrix matched calibrations.

Calibration curves were built at eight different mycotoxins levels, from LOQ to 100 times LOQ (from 0.1 to 300 ppm). Each level was prepared in triplicate. Slopes of standard solutions were compared with those obtained in matrix matched standards to assess the possible matrix effect on the chromatographic response. The results obtained showed that the matrix effect calculated for AFB_1_ and AFB_2_ were of 87.4 and 89.6% respectively.

**Figure 4:**
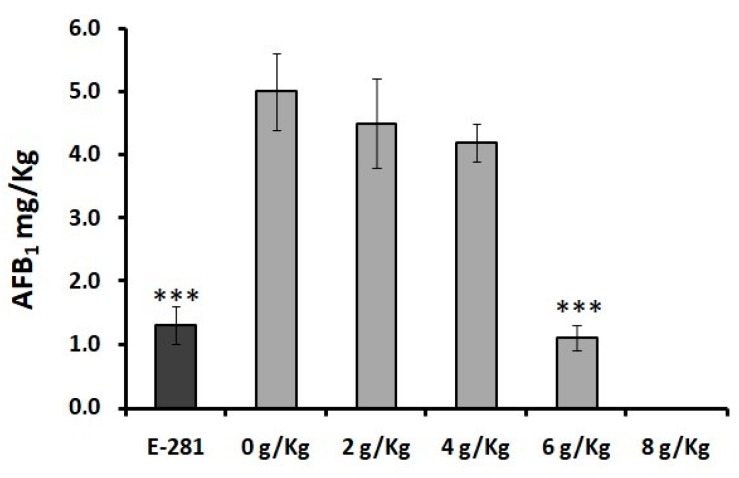
Concentration and reduction rate of AFB_1_ present in bread loaves contaminated with *A. flavus* and treated with several preserving treatments (Black = synthetic commercial E-281 preservative, Grey = Different doses of natural YMF) at 10 days of incubation. Significantly different from the bread without treatment, *p* ≤ 0.05 (*), *p* ≤ 0.001 (**), *p* ≤ 0.0001 (***).

The analysis of AFs in the bread inoculated with *A. flavus* (Figure 4), evidenced only the production by the mycotoxigenic fungi of the AFB_1_. Analyzing the results shown in the figure, the breads produced with 2, 4 and 6 g/kg of YMF and contaminated with *A. flavus*, presented amounts of the AFB_1_ not different from the statistical point of view in comparison with the control experiment, whereas in bread loaves treated with 8 g/kg of YMF no AFB_1_ concentration was detected, confirming the antifungal potential of the ingredient employed.

The amount of the AFB_1_ detected in the control experiment (a loaf of bread produced without any preservative ingredient) was of 5 mg/kg, whereas the loaves treated with sodium propionate showed a level of contamination of AFB_1_ of 1.3 mg/kg. The bread loaves treated with 6 g/kg of YMF presented 1.1 mg/kg of the AFB_1_ with a percentage of reductions of AFB_1_ in comparison with the control of the 78%. Mycotoxin production by *P. nordicum* was not observed in bread loaf contaminated with this fungus.

Saladino et al. [22] used ITCs derived from OMF and YMF (0.1, 0.5 and 1 g of flour) to avoid the production of AFs in piadina (a typical Italian flatbread) contaminated with *A. parasiticus*. In addition, the antifungal activity of the isothiocyanates toward *A. parasiticus* was also evaluated. The mustard flours employed in this study inhibited the growth of *A. parasiticus*, reducing the mycelium size by 12.2–80.6%. The ITC produced in situ also reduced the AFs biosynthesis in Italian piadina. The use of YMF reduced the AFs content by 41–69.2%. The same authors [23] investigated the use the ITCs generated by the addition of water to OMF and YMF for the reduction of the formation of mycotoxins produced by strains of the genus *Penicillium* as *P. expansum*. The patulin reduction (PAT) evidenced in the treated samples varied from 80 to 100%.

Hontanaya et al. [24] studied the reduction of AFs produced by *A. parasiticus* in nuts using ITCs (AITC and p-hydroxy benzyl isothiocyanate (p-HBITC)) produced by the enzymatic hydrolysis of glucosinolates sinigrin (SG) and sinalbin (SA) present in OMF and YMF. The reduction of AFB_1_, B_2_, G_1_ and G_2_ observed ranged from 83.1 to 87.2% and from 27.0 to 32.5% after nuts exposure to AITC and p-HBITC respectively.

### 2.4. Shelf Life Analysis

Considering the results of the shelf life improvement of the bread loaves treated with the sodium propionate and with different concentrations of YMF, it’s possible to underline several important data. 

In particular, considering the results on the mycotoxigenic fungi *A. flavus*, the control bread (bread produced with any preservative compound or ingredient) showed a visible fungal growth at 2 days of incubation (Table 3a), whereas the bread treated with sodium propionate (E-281), presented an evidence of the fungal growth at 3 days of incubation. The bread loaves produced with 2 and 4 g/kg of the YMF presented the same pattern of the *A. flavus* growth recorded for the experiment carried out with the E-281. The bread loaves produced with 8 g/kg of YMF, did not shown any fungal growth during the incubation period used in this study.

**Figure 5:**
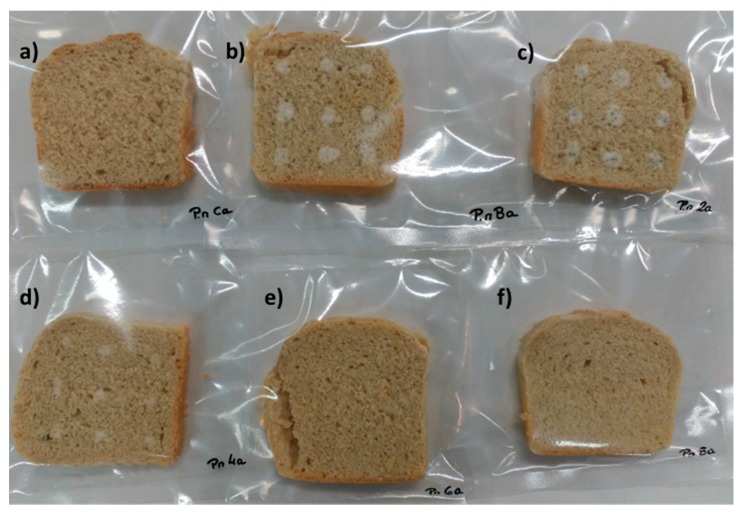
Bread loaves contaminated with *P. nordicum* and treated with (**a**) 2.0 g/kg of sodium propionate (E-281); (**b**) not treated; (**c**) 2 g/kg of YMF; (**d**) 4 g/kg of YMF; (**e**) 6 g/kg of YMF and (**f**) 8 g/kg of YMF, after 10 days of incubation.

Considering the shelf life improvement observed in the bread loaves contaminated with *P. nordicum* and treated with the antimicrobial ingredients tested (Table 3b), the bread produced with E-281, presented a visible fungal growth at 5 days of incubation, while the control bread and the loaves produced with 2 and 4 g/kg of YMF, presented a microbial growth at 2 and 4 days respectively. In the breads baked using 6 and 8 g/kg of YMF, it was not observed any *P. nordicum* growth during the incubation period used (Figure 5).

## 3. Materials and Methods

### 3.1. Chemicals and Microorganisms

AFs B_1_, B_2_, G_1_, G_2_, sinigrin (98% purity), formic acid (HCOOH), sodium propionate, tetrabutylammonium hydrogen sulfate (TBA), ammonium formate, and sodium chloride (NaCl) were obtained from Sigma Aldrich (St. Louis, MO, USA). YMF and OMF were provided by G.S. Dunn Dry Mustard Millers (Hamilton, ON, Canada). Methanol was purchased from Fisher Scientific (Rockingham County, NH, USA). Deionized water (<M18 MX cm resistivity) was obtained from a Milli-Q water purification system (Millipore, Bedford, MA, USA). Chromatographic solvents and water were degassed for 20 min using a Branson 5200 (Branson Ultrasonic Corp., Danbury, CT, USA) ultrasonic bath. The strains of *Aspergillus parasiticus* CECT 2681, *Penicillium camemberti* CECT 2267, *Penicillium roqueforti* CECT 2905, *Penicillium nordicum* CECT 2320, *Penicillium commune* CECT 20767, *Penicillium brevicopactum* CECT 2316, *Pencillium expansum* CECT 2278, *Penicillium chrysogenum* CECT 2668, *Penicillium solitum* CECT 20818, *Penicillium digitatum* CECT 2954 and *Fusarium graminearum* CECT 20486 were obtained from the the Spanish Type Culture Collection (CECT, Valencia, Spain). The strains of *Aspergillus flavus* ISPA 8111, *Aspergillus carbonarius* ISPA 5010 and *Fusarium verticiloides* ISPA 12044 were obtained from the Institute of Sciences of Food Production (ISPA—CNR, Bari, Italy). Buffered peptone water, potato dextrose agar (PDA) and potato dextrose broth (PDB) were acquired from Oxoid (Madrid, Spain).

### 3.2. Extraction of the Water-Soluble Components from Mustard Flours

The water-soluble components of YMF and OMF were extracted using the method of Hontanaya et al. [24] with some modifications. Flour (5 g) was placed in a 50 mL glass tube and autoclaved at 115 °C for 15 min to inactivate the enzyme myrosinase. This matrix was used to confirm that the possible antimicrobial activity of the mustard flours was due to the ITCs. Experiments with untreated mustard flours were carried out directly using the flour matrices. 25 mL of distilled water was introduced into the same tube and the extraction was carried out using an UltraTurrax T18 basic mixer (Ika, Staufen, Germany) for 3 min at 11,000 rpm. The mixture was centrifuged at 2500 rpm for 5 min at 4 °C and filtered through a filter paper (Whatman No. 4) in 50 mL tubes with screw cap. Finally, the extract was filtered again through a 0.22 μM filter and kept under refrigeration at 4 °C. To test also the antimicrobial activity of the concentrated mustard extracts, half of them were lyophilized by depositing 25 mL of extract in 100 mL plastic containers in a Virtis SP SCIENTIFIC Sentinel 2.0 lyophilizer (Warminster, PA, USA).

### 3.3. Antifungal Activity Tests on Solid Medium

The method used to evaluate the antimicrobial activity of the components of water-soluble mustard meal was as follows. The lyophilized extracts of YMF and OMF were previously suspended in 1 mL of sterile water obtaining a concentrated extract. The fungi to be analyzed were grown on the surface with a cotton swab in 9 mm Petri dishes prepared with 20 mL of PDA. In each plate, 10 μL in surface and 100 μL in pre-prepared agar wells were added. The triplicates of each plate were incubated at 28 °C observing the development of fungal growth at 48 h.

### 3.4. Antifungal Activity Tests on Liquid Medium

The assay was performed in 96-well sterile microplates, using the modified method of Siah et al. [25]. The tests of antifungal activity in liquid medium were not carried out with OMF due to the results obtained in the tests in solid medium. The first column served as control of the medium. For this, 200 μL of PDB were added in each of the wells to verify the absence of contamination of the medium. The next column consisted of a control of the microorganism, to verify its viability, so 100 μL of PDB was deposited. The remaining (3–12) contained 100 μL of lyophilized YMF extract resuspended at doses between 30 to 15,000 ppm. Each well was inoculated with 100 μL of a 5 × 10^4^ spores/mL suspension in PDB of the mycotoxigenic fungi described in the paragraph 2.1. The negative control consisted of inoculated medium without any treatment. The plates were incubated at 25 °C for 72 h in the dark. Four wells were used for each assay of each fungus (2 fungi per 96-well plate) and the experiments were performed in triplicate.

The minimum inhibitory concentration (MIC) was defined as the lowest concentration of the YMF extract, where the fungi did not show any visible growth. For the determination of the minimum fungicidal concentration (MFC), after determining the MIC, the concentrations corresponding to the inhibitory and to higher concentrations, as well as the controls, were subcultured on PDA plates. After 72 h of incubation at 25 °C, MFCs’ readings were made being MFC the lowest extract concentration in which a visible growth of the subculture was prevented.

### 3.5. Baking and Bread Treatment

The bread recipe included 600 g of wheat flour, 20 g of sucrose, 10 g of NaCl, 40 g of yeast for bakery products (Levital, Spain) and 250 mL of tap water. The commercial control was prepared adding to the ingredients sodium propionate (E-281) at 2 g/kg that is a common preservative used for loaf bread production in Spain. The studied breads were produced adding to the basic ingredients YMF at the concentration of 0, 2, 4, 6 and 8 g/kg of dry ingredient in the dough. The ingredients were kneaded manually for 10 min and the dough produced was left rising for 1h at room temperature. Baking was performed at 230 °C for 30 min in a deck oven (MIWE, Arnstein, Germany). The oven was pre-steamed (300 mL of water) before cooking. The loaves were kept for 30 min on cooling racks at room temperature. Loaves were cut in slices of 30 g each. The slices were inoculated with 100 µL of a suspension containing 1 × 10^5^ conidia/mL of *A. flavus* or *P. nordicum*. Conidial concentration was measured by optical density at 600 nm and adjusted to 10^5^ conidia/mL in PDB as reported by Kelly et al. [26] and introduced in 1 L plastic trays. All plastic trays were closed hermetically and incubated at room temperature for 10 days. Each day until the analysis the bread slices were examined to determine the visible fungal growth and the shelf life evaluation. Then, all packages were opened, and samples contaminated with *A. flavus* were used to determine the AFB_1_ content by liquid chromatography coupled to mass spectrometry in tandem (LC-MS/MS). Each bread was made in triplicate and from each bread 3 slices were analyzed (n = 9).

### 3.6. Aflatoxins Extraction

AFs extraction was performed using the method described by Hontanaya et al. [24] Briefly, the bread slices were finely grounded with a blender (Oster Classic grinder, Oster, Valencia, Spain) and 5 g samples were placed in a 50 mL plastic tube. Then, 0.5 g of sodium chloride (NaCl) and 25 mL of a methanol/water (80:20, *v*/*v*) mixture were added. Samples were homogenized using an Ultra Ika T18 basic UltraTurrax (Staufen, Germany) for 3 min. The mixture was centrifuged at 4500 rpm for 5 min and the supernatant was evaporated to dryness with a Rotavapor R-200 (Büchi, Postfach, Switzerland). The residue was re-dissolved in 1 mL of extraction solvent, filtered through a 0.22 µm syringe filter and injected to the LC-MS/MS system.

Commission Decision 2002/657/EC [27] and 401/2006/EC [21] were used as guidelines for the validation studies. All the parameters were evaluated by spiking blank samples (5, 10 and 15 μg/kg of AFB_1_ and AFB_2_), which were left to equilibrate overnight before the analysis. For identification purposes, retention time of compound in standards and samples were compared at tolerance of 70.5%. Method performance characteristics such as linearity, limits of detection (LOD), limits of quantitation (LOQ), matrix effect, recovery, repeatability and reproducibility were evaluated for sinigrin.

### 3.7. AFs Identification and Quantification by LC-MS/MS

LC-MS/MS analyses were performed with a system consisting of Agilent 1200 chromatograph (Agilent Technologies, Palo Alto, CA, USA) coupled to a 3200 QTRAP mass spectrometer (Applied Bio-systems, AB Sciex, Foster City, CA, USA) equipped with a turbo ionspray electrospray ionisation (ESI) interface. The instrument data were collected and processed using the Analyst version 1.5.2 software (AB Sciex, Foster City, CA, USA). Separation of analytes was performed using a reversed-phase analytical column (Gemini C18 column, 150 × 2 mm, I.D. 3 µm particle size), equipped with a security guard cartridge C18 (4 × 2 mm, I.D.; 5 µm) all from Phenomenex (Madrid, Spain). The mobile phases used were: Water with 0.1% of formic acid and 5 mM ammonium formate (Phase A) and methanol with 0.1% of formic acid and 5 mM ammonium formate (Phase B). The elution gradient was established initially with 10% eluent B. It was increased to 80% in 1.5 min and was kept constant until 4th min. Then it increased to 90% for 6 min. Subsequently, it was increased to 100% until the 14th minute and then reduced to 50% at the minute 17. Afterwards the initial conditions were maintained for 5 min. The flow rate was 0.25 mL min and MS/MS were achieved in the selected reaction monitoring (SRM) mode using ESI in positive mode. For LC-MS/MS analysis, scheduled SRM was used with a 120 s SRM detection window and 1 s of target scan time. The applied parameters were: ion spray voltage, 5500 V; source temperature,450 °C; curtain gas, 20; ion source gas 1 (sheath gas), 50 psi; ion source gas 2 (drying gas), 55 psi. Nitrogen served as nebulizer and collision gas. The ionization and fragmentation parameters used for the detection and quantification of the AFs were set according to Liu et al [16].

### 3.8. Determination of the Fungal Population

After incubation, each slice was weighed and transferred to a sterile plastic bag with sterile peptone water (Oxoid) in a 1:10 dilution and homogenized with a stomacher (IUL, Barcelona, Spain) during 30 s. The mixture was serially diluted in sterile plastic tubes containing 0.1% peptone water. Aliquots of 100 µl were seeded in acidified PDA (pH 3.5) (Insulab, Valencia, Spain) and the plates were incubated at 25 °C for 7 days before the microbial count [17].

## 4. Conclusions

The present study demonstrated the capacity of YMF extracts to inhibit or reduce the growth of several fungal strains belonging to *Aspergillus*, *Penicillium* and *Fusarium* species. Moreover, YMF used as an ingredient in bread preparation can significantly retard and reduce *A. flavus* and *P. nordicum* growth, improve the shelf life of the bread and reduce AFB_1_ naturally produced on substrate by *A. flavus*, so this study shows that YMF can be potentially used as natural preservative for bakery products which are commonly contaminated by *Aspergillus* and *Penicillium* species. YMF could be a potential substitute to conventional preservatives used in bread satisfying the growing demand of the consumer for natural products free from common chemical preservatives.

## Figures and Tables

**Table 1:** Antifungal activity evidenced by no autoclaved and autoclaved YMF and OMF against mycotoxigenic fungi employed in this study (E = Direct water extract; L = Lyophilized water extract). Calculation of antifungal activity: 8 mm diameter clearing zone (+), 10 mm diameter clearing zone (++), and more than 10 mm diameter clearing zone (+++).

Strains	YMF		Autoclaved YMF		OMF		Autoclaved OMF	
	E	L	E	L	E	L	E	L
*P. camemberti* (CECT 2267)	-	+	-	+	-	-	-	-
*P. roqueforti* (CECT 2905)	-	+	-	+	-	+	-	-
*P. nordicum* (CECT 2320)	-	+	-	-	-	-	-	-
*P. commune* (CECT 20767)	-	+	-	-	-	-	-	-
*P. brevicopactum* (CECT 2316)	-	+	-	-	-	-	-	-
*P. expansum* (CECT 2278)	-	+	-	-	-	-	-	-
*P. chrysogenum* (CECT 2668)	-	+	-	-	-	-	-	-
*P. solitum* (CECT 20818)	-	+	-	-	-	-	-	-
*P. digitatum* (CECT 2954)	-	+	-	-	-	-	-	-
*A. parasiticus* (CECT 2681)	+	+	-	-	-	-	-	-
*A. flavus* (ISPA 8111)	-	+	-	-	-	-	-	-
*A. carbonarius* (ISPA 5010)	-	+	-	-	-	+	-	-
*F. verticiloides* (ISPA 1044)	-	+	-	-	-	-	-	-
*F. graminearum* (CECT 20486)	-	-	-	-	-	-	-	-

**Table 2:** MIC and MFC evidenced by the YMF lyophilized water extract in the mycotoxigenic fungi tested.

Strain	MIC	MFC
	µg/mL	
*P. camemberti* (CECT 2267)	238.2	1906.2
*P. roqueforti* (CECT 2905)	476.5	1906.2
*P. nordicum* (CECT 2320)	937.5	1875.5
*P. commune* (CECT 20767)	937.5	1875.5
*P. brevicopactum* (CECT 2316)	937.5	1875.5
*P. expansum* (CECT 2278)	1875	7500.2
*P. chrysogenum* (CECT 2668)	937.5	3750.5
*P. solitum* (CECT 20818)	3750	3750.5
*P. digitatum* (CECT 2954)	937.5	7500.3
*A. parasiticus* (CECT 2681)	>15,000	>15,000
*A. flavus* (ISPA 8111)	>15,000	>15,000
*A. carbonarius* (ISPA 5010)	>15,000	>15,000
*F. verticiloides* (ISPA 1044)	1875.5	3750.5

**Table 3:** Shelf life, monitored in days, of the bread loaves treated with 4 different concentrations of the YMF and contaminated with (**a**) *Aspergillus flavus* (ISPA 8111) and (**b**) *Penicillium nordicum* (CECT 2320), in comparison with the commercial control produced with the additive E-281, and with a loaf bread prepared without any antimicrobial treatment.

(**a**)										
**Treatment**	**Days**									
	**1**	**2**	**3**	**4**	**5**	**6**	**7**	**8**	**9**	**10**
E-281	−	−	+	+	+	+	+	+	+	+
YMF 0 g/kg	−	+	+	+	+	+	+	+	+	+
YMF 2 g/kg	−	−	+	+	+	+	+	+	+	+
YMF 4 g/kg	−	−	+	+	+	+	+	+	+	+
YMF 6 g/kg	−	−	-	+	+	+	+	+	+	+
YMF 8 g/kg	−	−	−	−	−	−	−	−	−	−
(**b**)										
**Treatment**	**Days**									
	**1**	**2**	**3**	**4**	**5**	**6**	**7**	**8**	**9**	**10**
E-281	−	−	−	−	+	+	+	+	+	+
YMF 0 g/kg	−	+	+	+	+	+	+	+	+	+
YMF 2 g/kg	−	+	+	+	+	+	+	+	+	+
YMF 4 g/kg	−	−	−	+	+	+	+	+	+	+
YMF 6 g/kg	−	−	−	−	−	−	−	−	−	−
YMF 8 g/kg	−	−	−	−	−	−	−	−	−	−
